# Case Report: Heterotopic Pancreatic Tissue Leading to Acute on Chronic Cholecystitis

**DOI:** 10.7759/cureus.50192

**Published:** 2023-12-08

**Authors:** Yunus B Tekin, Michael S Anderson, Alexandra C Rhodes, Oscar F Hernandez, Elia Charbel Abboud

**Affiliations:** 1 General Surgery, Burrell College of Osteopathic Medicine at New Mexico State University, Las Cruces, USA; 2 General Surgery Specialists, Eastern New Mexico Medical Center, Roswell, USA; 3 Clinical Education, Burrell College of Osteopathic Medicine at New Mexico State University, Las Cruces, USA

**Keywords:** choledocholithiasis, laparoscopic, gallbladder, cholecystitis, ectopic pancreatic tissue, heterotopic pancreatic tissue

## Abstract

Heterotopic (ectopic) pancreatic tissue refers to tissue located outside the borders of the main pancreas. It is rarely found in gallbladders and can cause biliary disease, with only a few documented cases in the surgical literature. Here, we present the unusual case of a 21-year-old female with acute on chronic cholecystitis caused by obstruction of the cystic duct with ectopic pancreatic tissue. The aim of the paper is to describe the significance of ectopic pancreatic tissue in biliary pathology and bring awareness to clinicians about this rare entity.

## Introduction

There are many speculations regarding the origin of heterotopic pancreatic tissue. The most accepted theory is that heterotopic pancreas is formed from a lateral budding of the rudimentary pancreatic duct which penetrates the intestinal wall and is then carried by the longitudinal growth of the intestines. Another theory speculates that the pancreatic tissue is separated from the main pancreas during embryonic rotation [1,2]. Lastly, a more recent theory states that abnormalities in the notch signaling system during embryogenesis may contribute to heterotopic pancreatic tissue in the gallbladder [2].

Previous studies have shown that 90% of ectopic pancreatic tissue is found in the stomach, duodenum, and jejunum [3]. Of these, the stomach is the common location, accounting for 25%-38.2% [4]. While rare, they can be found in the gallbladder, as well as in a Meckel's diverticulum. Most of these findings are incidental, occurring in one out of 500 abdominal explorations and 2% of autopsies [3]. Men are three times more likely to have heterotopic pancreatic tissue than women are [5]. However, it has been found that there is a higher incidence of heterotopic pancreatic tissue in gallbladder cases in women [5]. 

Heterotopic pancreatic tissue can be broken down into four different types of tissue, which were defined by Heinrich in 1909, then later modified by Fuentes in 1973. Type 1 consists of typical pancreatic tissue with acini, ducts, and islet cells. Type 2 consists of canalicular variants with pancreatic ducts only. Type 3 consists of exocrine pancreatic tissue with acinar tissue only. Type 4 consists of endocrine pancreatic tissue with islet cells only [6].

The most common complaint associated with heterotopic pancreatic tissue is abdominal pain [6]. This pain could be due to a multitude of factors, such as local irritation and inflammation, release of pancreatic hormones and enzymes, and obstructive symptoms [6].

This article was previously presented as a poster at the 2022 Virtual Bioscience Poster Session, hosted by the Medical Center of the Americas, in El Paso, Texas on February 22, 2022 and as a poster at the Minimally Invasive Surgery Week (MIS), hosted by the Society of Laparoscopic & Robotic Surgeons, in New Orleans, Louisiana on September 8, 2022.

## Case presentation

Our patient is a 21-year-old female who presented to the emergency room with pain in the right upper quadrant and epigastric region, radiating to her back, that is stabbing and cramping in quality, with nonbloody, nonbilious vomiting, all of which have been worsening over the past seven days. Similar symptoms have occurred during her pregnancies in 2018 and 2019. She has also had a previous common bile duct stent placement, which was likely done for choledocholithiasis, in 2019. The stent was intended to remain in place for three months, but she did not follow up for removal.

**Figure 1:**
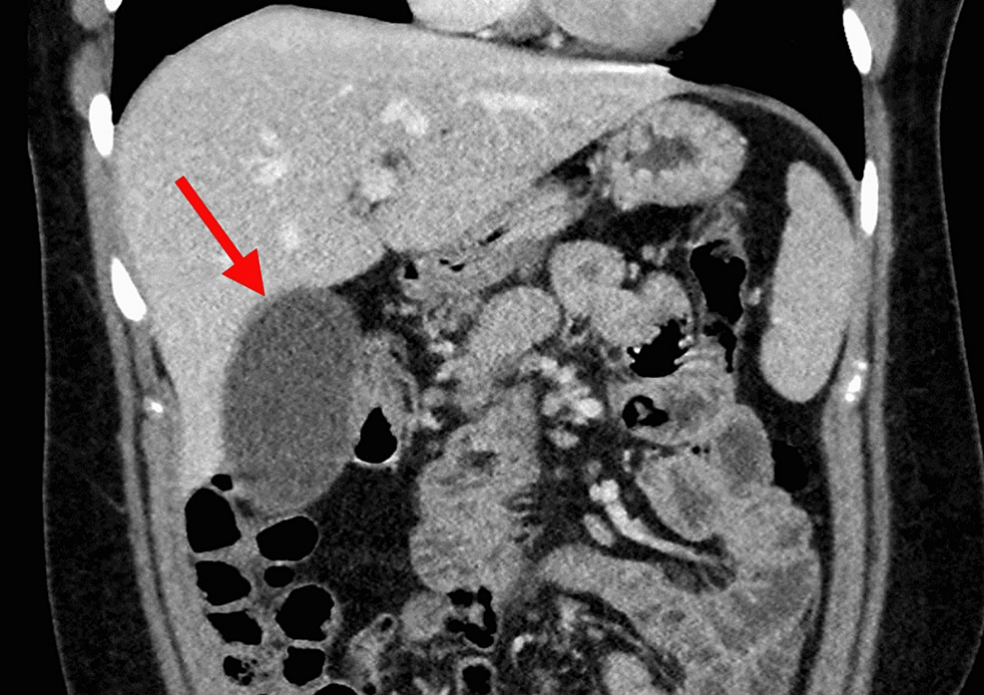
CT scan showing a distended and inflamed gallbladder. CT: computed tomography

Vital signs in the emergency room were non-significant including lack of fever. She had a right upper quadrant tenderness with a positive Murphy's sign. She showed no signs of peritonitis, guarding, or rebound tenderness. Initial labs showed pertinent findings of mildly elevated aspartate aminotransferase (AST), alanine aminotransferase (ALT), and alkaline phosphatase, with normal lipase and amylase values (Table 1). White blood cell count was normal (Table 2). CT without contrast of the abdomen showed a dilated and enhancing gallbladder with choledocholithiasis and common bile duct stent. No cholelithiasis or free air within the biliary system was visualized (Figure 1).

**Table 1:** Complete metabolic panel in September 2021 H: high, L: low, BUN: blood urea nitrogen, GFR: glomerular filtration rate, AST: aspartate aminotransferase, ALT: alanine aminotransferase

Chemistry	Value	Normal Range
Sodium	137	135-145 mEq/L
Potassium	4.2	3.5-5.3 mEq/L
Chloride	104	98-112 mEq/L
Carbon dioxide	22H	24-31 mEq/L
Anion gap	15.2	5-17
BUN	10	8-21 mg/dL
Creatinine	0.5L	0.6-1.3 mg/dL
Estimated GFR/1.73m2	>90	>60
Random glucose	84	65-105 mg/dL
Calcium	9.2	8.4-10.2 mg/dL
Total bilirubin	0.6	0.2-1.3 mg/dL
AST	79H	10-42 IU/L
ALT	136H	10-40 IU/L
Alkaline phosphatase	99H	32-85 U/L
Total protein	7.6	6-8 gm/dL
Albumin	4.4	3.5-5.0 gm/dL
Amylase	63	12-125 U/L
Lipase	41	7-58 U/L

**Table 2:** Complete blood count in September 2021 H: high, L: low, WBC: white blood cell, RBC: red blood cell, HgB: hemoglobin, Hct: hematocrit, MCV: mean corpuscular volume, MCH: mean corpuscular hemoglobin, MCHC: mean corpuscular hemoglobin concentration, RDW: red cell distribution width

Hematology	Value	Normal Range
WBC	7.6	4.5-11.0 K/mm3
RBC	5.2	4.0-5.2 M/mm3
HgB	12.7	12-16 gm/dl
Hct	40.6	36-46 %
MCV	77.8 L	80-100 fl
MCH	24.3 L	26-34 pg
MCHC differential	31.3 L	33-36 g/dl
RDW	13.8	11.5-15.3 %
Platelet count	236	130-400 K/mm3
Neutrophils	62.3	40-76%

**Figure 2:**
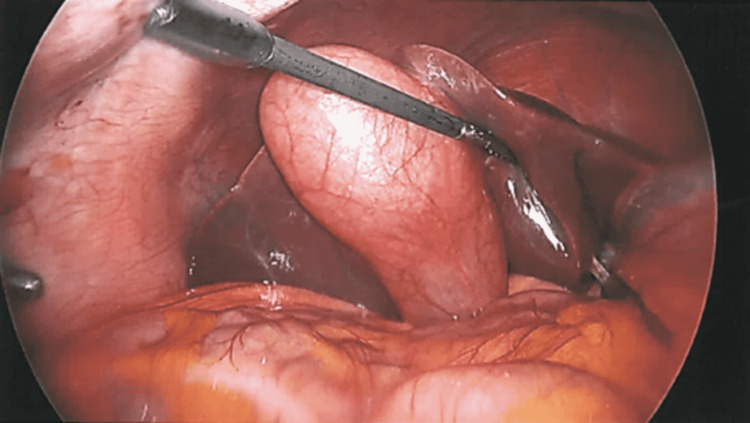
Distended and inflamed gallbladder (laparoscopic view).

**Figure 3:**
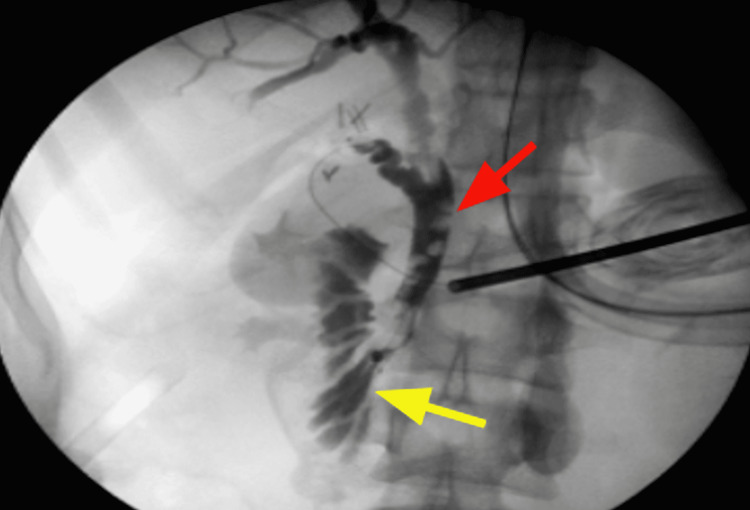
Intraoperative cholangiogram: choledocholithiasis (red arrow), patent CBD stent with filling of the duodenum (yellow arrow). CBD: common bile duct

Patient opted to proceed with surgical removal of her gallbladder. We performed a laparoscopic cholecystectomy with intraoperative cholangiogram. The gallbladder was inflamed, distended, and hydropic, and appeared to have a gallstone impacted at its neck (Figure 2). A critical view of safety was obtained with visualization of two, and only two, structures attached to the gallbladder, and clear visualization of the lower third of the cystic plate. The cystic artery was then clipped (two clips distally, one proximally) and ligated. Intraoperative cholangiogram showed non-obstructing choledocholithiasis and a patent CBD stent with filling of the duodenum (Figure 3). Right and left hepatic ducts showed normal filling. There was no contrast extravasation. The cystic duct was then clipped twice proximally to the ductotomy and ligated. Gallbladder was then carefully dissected away from the liver and removed via endoscopic retrieval bag.

**Figure 4:**
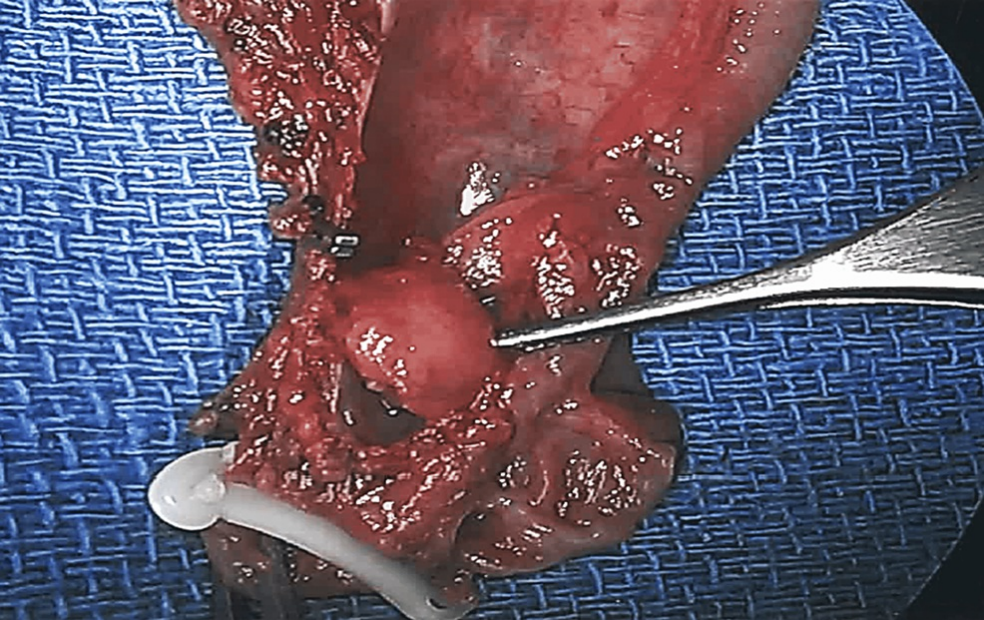
Submucosal lesion obstructing the orifice of the cystic duct.

Subsequent evaluation of the specimen on the back table revealed a submucosal lesion obstructing the cystic duct at its junction with the gallbladder (Figure 4). There were no stones visualized in the specimen. 

**Figure 5:**
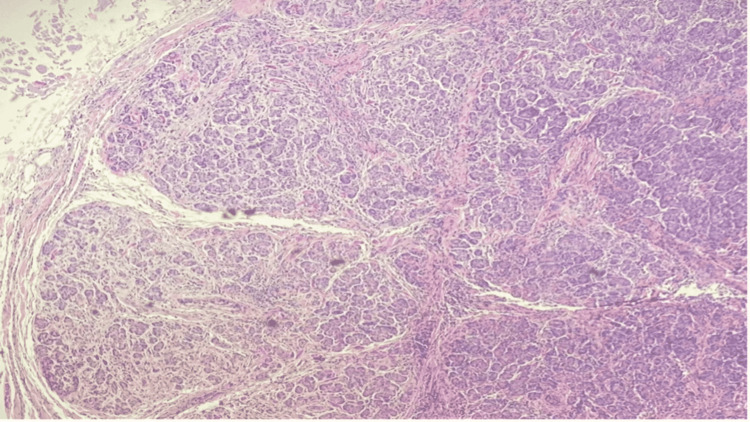
Type 3 heterotopic pancreatic tissue; acinar tissue only (low magnification).

**Figure 6:**
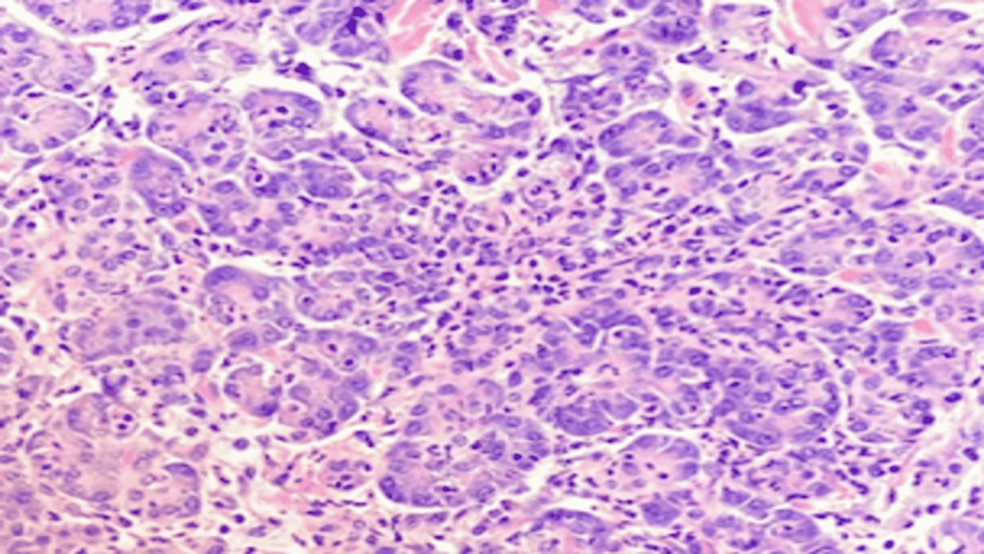
Type 3 heterotopic pancreatic tissue; acinar tissue only (high magnification).

The specimen was sent to pathology and further evaluation indicated acute and chronic cholecystitis. The gallbladder measured 9.0 cm in length and 3.1 cm in diameter with a thickness of 0.1 cm. The submucosal lesion revealed acute and chronic inflammation of heterotopic pancreatic tissue. The heterotopic pancreatic consisted of acinar tissue only (Figures 5, 6). This classifies as Type 3 ectopic pancreatic tissue (Table 3). Pathology results found no calculi within the gallbladder or the container.

**Table 3:** Heterotopic pancreatic tissue classifications. As defined by Heinrich in 1909, then later modified by Fuentes in 1973 [6]

Classification	Characteristics
Type 1	Typical pancreatic tissue with acini, ducts, and islet cells
Type 2 (canalicular variety)	Pancreatic ducts only
Type 3 (exocrine pancreas)	Acinar tissue only
Type 4 (endocrine pancreas)	Islet cells only

## Discussion

We speculate that our patient’s chronic biliary colic was caused by this lesion, with eventual complete obstruction of her cystic duct causing acute on chronic cholecystitis. Pathology confirmed acute and chronic cholecystitis. However, as was seen in our patient’s pathology findings, it is also possible for heterotopic pancreatic tissue to become acutely or chronically inflamed, and this pancreatitis can itself cause symptoms in the host organ. Researchers have also proposed that elevated amylase and lipase levels in the presence of gallbladder symptoms could be due to inflammation and damage caused by heterotopic pancreatic tissue [5]. Hyperinsulinism, acute pancreatitis and calcifications can also be observed [5]. Our patient did not have elevated pancreatic enzymes, amylase 63 (12-123U/L) and lipase 41 (7-58U/L). With our patient's normal serum pancreatic enzymes, it is unlikely that this was the cause of her acute symptoms. Moreover, our intraoperative findings of a hydropic gallbladder are most consistent with prolonged obstruction of the cystic duct. It has been noted by Elpek et al. that heterotopic pancreatic tissue in the neck region of the gallbladder prevented bile leak and caused hydrops of the gallbladder [2]. In another case, heterotopic pancreatic tissue caused perforation of the gallbladder, leading to peritonitis [2].

Due to endoscopic limitations in our rural community hospital, the patient had to be referred out for retrieval of the common bile duct stones and removal of the stent. We speculate these stones are cholesterol predominate due to the patient's weight, recent pregnancies, and no history of hemolysis or infection. It is possible that our patient's heterotopic lesion was not obstructing initially, which led to the passing of gallstones. However, sometime between when the common bile duct stent was placed and her presentation to our hospital, her heterotropic pancreatic tissue became inflamed, enlarged, and obstructed her cystic duct.

The findings of this paper raise many questions about the significance of heterotopic pancreatic tissue. Patients should not be alarmed by this finding, as it is most often benign. However, previous studies have shown that heterotopic pancreatic tissue can turn malignant. Researchers reported adenocarcinoma in two separate cases of heterotopic pancreatic tissue [7]. Just like in our case, heterotopic pancreatic tissue was shown to cause obstructions elsewhere in the body. There were two reported cases of ectopic pancreatic tissue causing gastric outlet obstruction, presenting as typical gastric outlet obstruction symptoms [4]. Other studies have also shown heterotopic pancreatic tissue causing bleeding, and the formation of cysts, abscesses, and polyps [4-6].

A clinical diagnosis of heterotopic pancreatic tissue is unlikely. This case report will not change the management of cholecystitis/choledocholiatiasis. The presentation can mimic typical cholecystitis symptoms and therefore must be treated as such. An ultrasound diagnosis of heterotopic pancreatic tissue causing obstructive symptoms of the gallbladder is challenging as it is nearly impossible to differentiate it from polyps, adenomas, and carcinoma [3]. Heterotopic pancreatic tissue can cause a multitude of pathologies in the gallbladder, as reported in this case. The only way to diagnose this rare pathological finding is through biopsy and pathology consultation.

## Conclusions

Although rare, heterotopic pancreatic tissue can be considered as a differential in the evaluation of biliary disease. This case demonstrates a rare cause of cholecystitis and highlights the importance of formulating a broad differential when evaluating a patient with biliary symptoms. Ultimately, however, standard surgical treatment for cholecystitis should be undertaken. From local inflammation to obstructive symptoms, heterotopic pancreatic tissue leading to cholecystitis can present in many different ways. Further research should be focused on assessing the different classifications of heterotopic pancreatic tissue and their clinical presentation and significance. 
